# The Alu-insertion progesterone receptor gene polymorphism is not associated with breast cancer: a meta-analysis

**DOI:** 10.1186/s12881-018-0529-5

**Published:** 2018-01-25

**Authors:** Jun Yao, Xing-ling Qi, Yong Zhang

**Affiliations:** 1 School of Forensic Medicine, China Medical University, No. 77 Puhe Road, Shenbei New District, Shenyang, 110122 People’s Republic of China; 2 Cancer Hospital of China Medical University, Liaoning Cancer Hospital & Institute, Shenyang, 110042 People’s Republic of China

**Keywords:** Progesterone receptor, Alu insertion, Breast cancer, Meta-analysis

## Abstract

**Background:**

The role of progesterone receptor (PGR) gene polymorphisms in breast cancer is still controversial. Here, we performed a meta-analysis to determine whether the Alu insertion is associated with an increased risk of breast cancer and, further, whether the Alu insertion contributes to the development of breast cancer.

**Methods:**

Using database searches, we selected 10 controlled case studies that met a rigorous set of inclusion criteria; these studies included 2106 cases and 1660 controls. We generated odds ratios and 95% confidence intervals in order to determine the strength of the relationship between the Alu insertion and breast cancer incidence. We also performed additional subgroup analyses and sensitivity analyses to further clarify the relationship.

**Results:**

Using a random effects model, we concluded that the Alu insertion was not associated with the risk of breast cancer under the dominant genetic model; the pooled OR was 1.025 (95% CI = 0.526–1.994, *p* = 0.943). When a subgroup analysis was performed according to ethnicity, we found that the Alu insertion was associated with breast cancer incidence in Indians and Indo-European mixed racial groups, but the association disappeared for patients of Caucasian or Latino decent.

**Conclusions:**

Our meta-analysis showed that the Alu-insertion progesterone receptor gene polymorphism was not associated with breast cancer. These results provide further information regarding the association between the Alu insertion in the *PGR* gene and the incidence of breast cancer.

## Background

Breast cancer is the leading cause of cancer-related deaths in women worldwide; globally, approximately 1 million women experience breast cancer each year [1, 2]. Current research demonstrates that an interaction between multiple abnormal genetic and environmental factors can generate a susceptibility to breast cancer [3]. A multitude of factors have been postulated to influence the development of breast cancer including age, ethnicity, early or delayed menarche, use of oral contraceptives, breastfeeding, and age at menopause as well as a number of genetic factors [4, 5]. Nevertheless, a definite etiology of breast cancer has not yet been identified.

The human progesterone receptor, encoded by the *PGR* gene, is a member of the steroid receptor superfamily. The progesterone receptor is essential for mediating the effects of progesterone, which is critical for the establishment and maintenance of pregnancy. Intracellular PGRs and their associated protein kinase C molecules are known to regulate tumor cell proliferation and metastasis, such as during the infiltration process of human glioblastomas [6]. Moreover, membrane PGR have been shown to mediate most non-classical progesterone actions; as such, membrane PGRs may be useful as pharmacologic targets or biomarkers of cancer and other reproductive diseases [7]. In addition, increased expression of PGRs has been reported in gastric cancer [8]. Signature patterns of expression of the estrogen and PGR signaling pathways may be used to predict prognosis and guide management of colorectal cancer [9]. Therefore, polymorphisms in *PGR* that effect its level of expression may be associated with variations in the risk of breast cancer. Several reports have attempted to address the relationship between PGR expression and breast cancer risk in populations of various ethnicities, although with mixed results [10–14].

The Alu element, a short interspersed nuclear element, is the most successful retrotransposon in primate genomes; it exists at an estimated copy number of 1.1 million in the human genome [15]. Each Alu element is approximately 300 bp in length and has a dimeric structure. Because of their abundance and sequence identity, Alu elements are frequently involved in genomic rearrangements within the human genome. Genomic rearrangements can result in genetic disorders such as Alport syndrome, Fabry disease, and peeling skin disease [16–19]. In fact, Alu elements are related to about 0.1% of human genetic disorders [20]. Recently a young human-specific 306 bp Alu insertion was found in the *PGR* gene, in intron G between exons 7 and 8. The Alu insertion contains a half-ERE/SPI site, which may disproportionately increase the transcription of PGR after estrogen stimulus [21]. Some studies reported that this particular Alu insertion was associated with breast cancer [22, 23], while others did not observe the association [24–26].

These conflicting results are likely due to the limited sample size found in the studies as well as differing genetic backgrounds. Meta-analysis is widely-used in medicine as a statistical method of reconciling studies with inconsistent results [27]. Therefore, we carried out a meta-analysis of studies investigating the relationship between the Alu insertion and the risk of breast cancer.

## Methods

### Selection and inclusion criteria for relevant studies

We searched three online electronic databases (Embase, PubMed, and Web of Science) in order to identify potential studies for inclusion in our meta-analysis; the data of the last search update was November 2016. We used the following key words in our literature searches: progesterone receptor, PROGINS, Alu insertion, breast cancer, and mammary. Articles cited by the potential studies and relevant review articles were also checked for additional supplementary studies. The following inclusion criteria were utilized: (1) a case-control study design; (2) patients with a diagnosis of breast cancer; and (3) inclusion of the allele and/or genotype frequencies. In cases where studies included the same or overlapping data, we chose the most recent article. The corresponding authors were contacted for other useful information (such as sample characteristics) and additional data not presented in the initial article.

### Data extraction

Two researchers (Jun Yao and Xing-ling Qi) independently collected the relevant data, from all eligible publications. If there was a disagreement, a consensus was obtained after discussion. The following characteristics were collected from each study chosen for inclusion: first author’s last name, publication year, region, ethnicity, numbers of each genotype of both cases and controls, and source of controls.

### Statistical analysis

Our meta-analysis was completed using Stata version 10.0 (Stata Corp., College Station, TX). We used a chi-square test to determine the Hardy-Weinberg equilibrium (HWE) of each genotype frequency in control subjects. Thakkinstian’s method was used to calculate pooled frequency analyses [28]. All statistical tests were two-sided and *p* < 0.05 was considered statistically significant.

In order to measure the strength of the association between breast cancer and the Alu insertion, we calculated odd’s ratios (ORs) with 95% confidence intervals (Cis). We used a random effects model to pool the effect sizes across studies. This model measure the possible effect size across populations with different genetic backgrounds after considering the heterogeneity among the included studies [29]. An allele contrast model, a dominant model, and a recessive model were used to calculate overall pooled ORs. Using A as the risk allele, we compared OR_1_ (AA vs. aa), OR_2_ (Aa vs. aa), and OR_3_ (AA vs. Aa); these pairwise differences were used to select the most appropriate genetic model under the instruction as previously described [27, 30].

We also determined the degree of heterogeneity across studies using the Q-statistic; *p* > 0.05 indicated a lack of heterogeneity and *p* < 0.05 indicated the presence of heterogeneity [31, 32]. I^2^ was the proportion of detected variation in effect size due to the actual discrepancies across studies; classically, the I_2_ statistic is used to define low (< 25%), moderate (~ 50%), and high (> 75%) levels of heterogeneity [33]. We also performed subgroup analyses by ethnicity (i.e. Caucasian, African-American, Latino, Indian, and Indo-European) as well as by source of control subjects (i.e. hospital-based vs. population-based).

We used a funnel plot to estimate publication bias. The standard error of log(OR) for each study was plotted vs. its log(OR); asymmetric plots indicated potential publication bias. The degree of asymmetry was measured using Egger’s test; *p* < 0.05 was considered significant publication bias [34].

Finally, we performed sensitivity analyses to measure the potential influence of each study on the final pooled effect size as previously described [35].

## Results

**Fig. 1:**
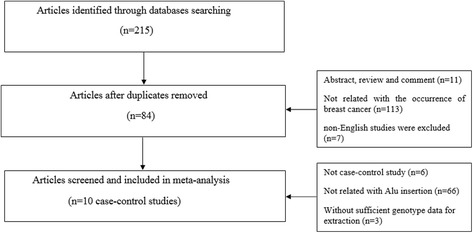
Study selection process in this meta-analysis

After excluding published works with overlapping data and those that did not meet inclusion criteria, the final meta-analysis included a total of nine published articles containing 10 studies including 2106 cases and 1660 controls [22–26, 36–39] (Fig. 1). Key characteristics of the included studies are described in Table 1. The frequencies of each genotype and allele along with their HWE values are listed in Table 2. Of the 10 studies, only one study showed significant deviation from the expected HWE (*p* = 0.0390) [23].

**Table 1:** Baseline characteristics of qualified studies in this meta-analysis

Author	Year	Country	Ethnicity	Controls source	Mean age of control group	Cases, n	Controls, n
Manolitsas [36]	1997	England	Caucasian	population-based	–	292	220
Lancaster [25]	1998	America	Caucasian	hospital-based	–	68	101
Wang-Gohrke [23]	2000	Germany	Caucasian	population-based	42.9	559	554
Fabjani [24]	2002	Austria	Caucasian	population-based	–	155	106
Donaldson [26]	2002	America	Caucasian	population-based	–	23	60
Donaldson [26]	2002	America	African-American	population-based	–	61	81
Linhares [37]	2005	Brazil	Latino	population-based	–	50	49
Romano [38]	2007	Netherlands	Caucasian	hospital-based	–	167	31
Surekha [39]	2009	India	Indian	population-based	–	250	249
Gallegos-Arreola [22]	2015	Mexico	Indo - European mixed race	population-based	53.64	481	209

**Table 2:** Distribution of genotype and allele frequencies of the *PGR* Alu insertion

Author	Genotype distribution						*P*_HWE_	Allele frequency			
	Cases, n			Controls, n				Cases, %		Controls, %	
	T_1_T_1_	T_1_T_2_	T_2_T_2_	T_1_T_1_	T_1_T_2_	T_2_T_2_		T_1_	T_2_	T_1_	T_2_
Manolitsas [36]	229	61	2	162	54	4	0.8375	88.9	11.1	85.9	14.1
Lancaster [25]	55	12	1	79	18	4	0.0390	89.7	10.3	87.1	12.9
Wang-Gohrke [23]	426	128	5	393	144	17	0.3945	87.7	12.3	83.9	16.1
Fabjani [24]	119	32	4	78	28	0	0.1172	87.1	12.9	86.8	13.2
Donaldson [26]	17	5	1	41	16	3	0.3965	84.8	15.2	81.7	18.3
Donaldson [26]	56	5	0	73	8	0	0.6401	95.9	4.1	95.1	4.9
Linhares [37]	31	18	1	31	17	1	0.4416	80.0	20.0	80.6	19.4
Romano [38]	123	41	3	22	7	2	0.2075	85.9	14.1	82.3	17.7
Surekha [39]	241	7	2	242	7	0	0.8220	97.8	2.2	98.6	1.4
Gallegos-Arreola [22]	360	103	18	176	33	0	0.2153	85.6	14.4	92.1	7.9

Note: *P*_HWE_, the *P* value of Hardy-Weinberg equilibrium test in the genotype distribution of controls; T1/T1, homozygotes without the 306-bp insertion; T1/T2, heterozygotes with the insertion; T2/T2, homozygotes with the insertion

We calculated the pooled frequencies of the Alu insertion in the control populations stratified by ethnicity. T_1_/T_1_ represents homozygote individuals lacking the 306 bp Alu insertion, T_1_/T_2_ represents heterozygotes with the insertion, and T_2_/T_2_ indicates homozygous individuals with the Alu insertion. The allelic frequency of the Alu insertion varied across ethnicities: the pooled T_2_ allele frequency was highest among Indians (17.7%, 95% CI = 17.3–18.2%), followed by Caucasians (14.6%, 95% CI = 10.5–18.8%), Latinos (14.1%, 95% CI = 14.0–14.1%), African-Americans (4.9%, 95% CI = 4.9–5.0%), and Indo-Europeans (1.4%, 95% CI = 1.4–1.4%). The overall pooled T_2_ allele frequency was 12.6% (95% CI = 7.4–17.8%).

**Fig. 2:**
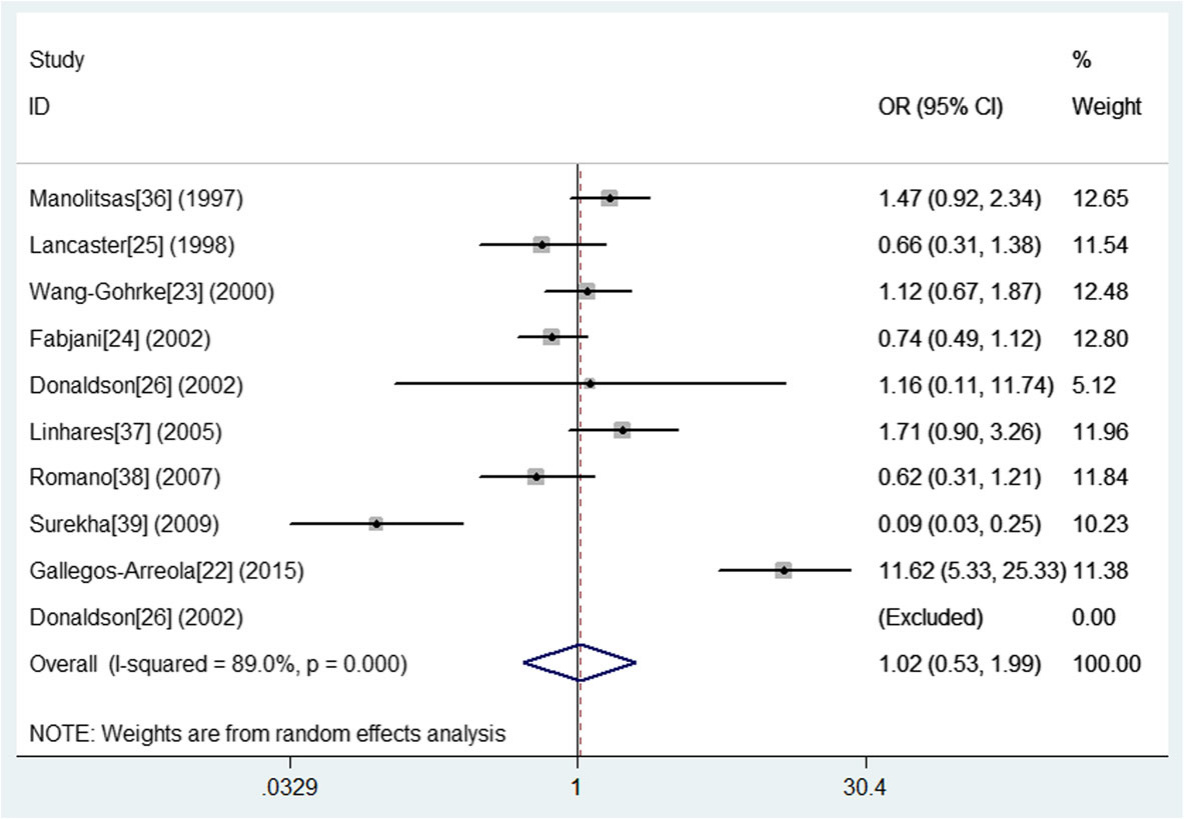
Forest plot of the association between the Alu insertion in the *PGR* gene and breast cancer in a dominant model (T_1_T_2_ + T_2_ T_2_ vs. T_2_ T_2_)

The association between the Alu insertion and the risk of breast cancer was determined in all 2106 cases and 1660 control subjects from 10 studies using pooled ORs and the corresponding 95% CIs for the homozygous codominant, heterozygous codominant, dominant, recessive, and allele contrast genetic models (Table 3). Finally, we selected the dominant model according to the principle of genetic model selection [30, 40]. The results indicated that there was no association between the Alu insertion and the occurrence of breast cancer (Fig. 2). For the dominant model, we used the random effects model to calculate a pooled OR of 1.025 (95% CI = 0.526–1.994, *p* = 0.943). Ethnicity subgroup analysis indicated that the Alu insertion was associated with breast cancer in Indian (OR = 0.091, CI = 0.033–0.254, *p* < 0.001) and Indo-European patients (OR = 11.620, 95% CI = 5.331–25.327, *p* < 0.001), but no association was found in Caucasians (OR = 0.916, 95% CI = 0.673–1.243, *p* = 0.576) or Latinos (OR = 1.712, 95% CI = 0.898–3.263, *p* = 0.102) (Table 4). Furthermore, we found no association between the Alu insertion and the risk of breast cancer in a subgroup analysis by source of controls (population-based: OR = 1.179, 95% CI = 0.515–2.699, *p* = 0.697; hospital-based: OR = 0.635, 95% CI = 0.385–1.046, *p* = 0.075).

**Table 3:** Summarized ORs with 95% CIs for the association between *PGR* polymorphism and breast cancer

Polymorphism	Genetic model	n	Statistical model	OR	95% CI	p_z_	I^2^(%)	p_h_	p_e_
Alu insertion									
	Allele contrast	10	Random	0.962	0.738–1.254	0.775	55.9	0.016	0.552
	Homozygous codominant	10	Random	0.948	0.325–2.763	0.922	58.7	0.013	0.132
	Heterozygous codominant	10	Random	1.019	0.535–1.939	0.955	87.3	< 0.001	0.776
	Dominant	10	Random	1.025	0.526–1.994	0.943	89.0	< 0.001	0.889
	Recessive	10	Random	0.948	0.440–2.042	0.892	48.5	0.042	0.389

Note: n, the number of studies; *p*_z_, *P* value for association test; *p*_h_, *p* value for heterogeneity test; *p*_e_, *p* value for publication bias test

**Table 4:** Stratified analysis of the association of *PGR* polymorphism with breast cancer under dominant model

Subgroup analysis	Alu insertion					
	n	OR	95% CI	p_z_	I^2^(%)	p_h_
Overall	10	1.025	0.526–1.994	0.943	89.0	< 0.001
Ethnicity						
Caucasians	6	0.916	0.673–1.246	0.576	35.0	0.174
African-American	1		excluded			
Latinos	1	1.712	0.898–3.263	0.102	–	–
Indians	1	0.091	0.033–0.254	< 0.001	–	–
Indo - European mixed race	1	11.620	5.331–25.327	< 0.001	–	–
Source of controls						
Population-based	8	1.179	0.515–2.699	0.697	91.1	< 0.001
Hospital-based	2	0.635	0.385–1.046	0.075	0.0	0.897

Note: n, the number of studies; *p*_z_, *p* value for association test; *p*_h_, *p* value for heterogeneity test

### Sensitivity analysis

Sensitivity analysis was conducted to measure the influence of each study on the resulting meta-analysis. The relevant pooled ORs indicated that no significant change appeared when each study was omitted, one at a time, from the overall meta-analysis. Thus, the final pooled results are both stable and reliable.

### Publication bias

**Fig. 3:**
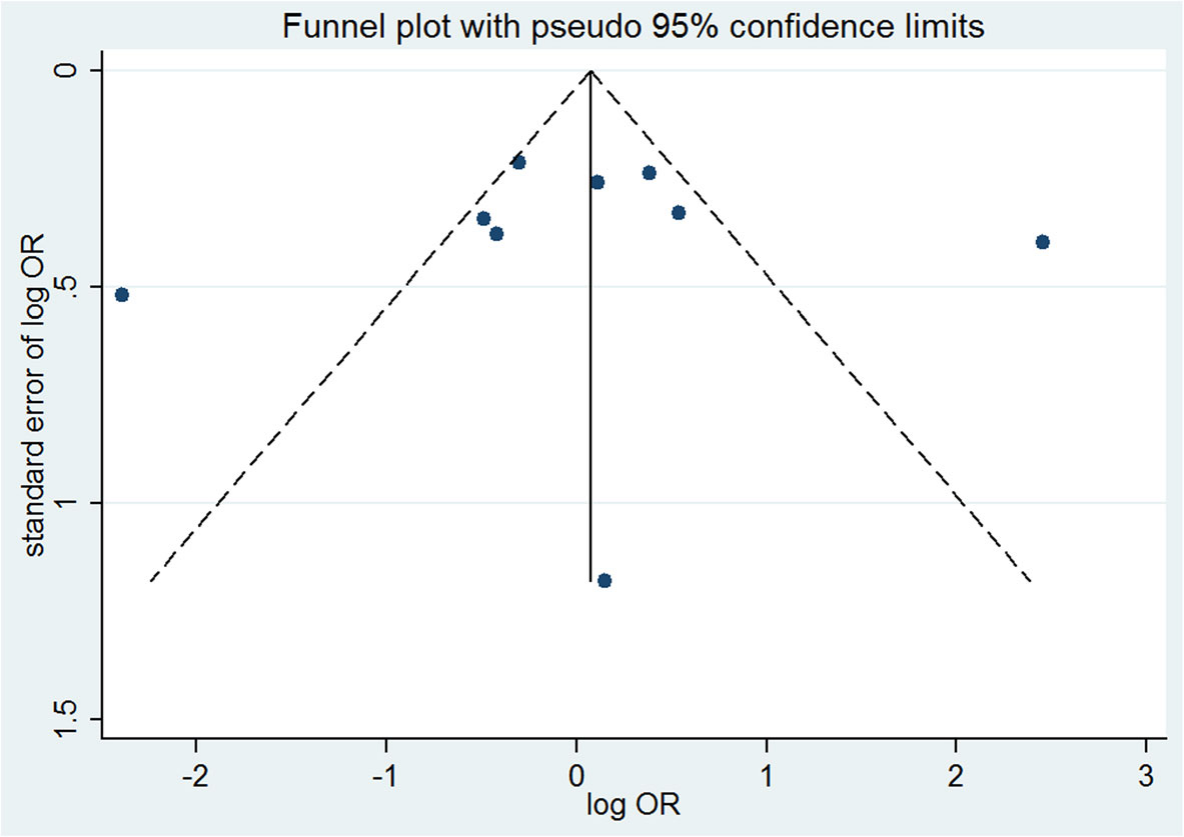
Funnel plot for evaluation of publication bias in breast cancer

The potential publication bias was evaluated using a funnel plot (Fig. 3). An Egger’s test was also utilized as a supplementary test of bias; results of this test also indicated no publication bias (*p* = 0.889).

## Discussion

Our meta-analysis included 10 studies, comprised of 2106 cases and 1660 controls, that investigated the association between *PGR* gene polymorphism (specifically, the Alu insertion) and the occurrence of breast cancer. Overall, the results of our meta-analysis provide evidence that the presence of the Alu insertion is not associated with an increased risk of breast cancer. Subgroup analyses by ethnicity or source of controls were used to investigate potential disequilibrium in the distribution of cases and controls. We also performed a sensitivity analysis, which reinforced the validity of the results.

Associations between *PGR* variation and breast cancer varied across different populations. The Alu insertion was related to breast cancer in both Indians and Indo-European mixed racial groups, while this association disappeared in Caucasians and Latinos. This difference in association is likely due to two factors. First, the various genetic backgrounds of the racial groups may contribute as the frequency of the Alu insertion varies across different populations. Certainly, genetic factors confer higher risk levels for breast cancer [41]. However, different populations also have different life-styles and are often influenced by different environmental factors [42]. In the end, the occurrence of breast cancer is determined by the interaction between genetic factors and the environment.

Although the exact biological role of the Alu insertion into the *PGR* gene is not yet clear, it has been reported that the insertion might cause abnormal gene transcription and weaken the binding of progesterone to the PCR, which would subsequently reduce the activity of progesterone [22]. Therefore, PGR dysfunction caused by the Alu insertion may potentially affect the occurrence of breast cancer.

Finally, there are several potential limitations of our study. First, there was measurable heterogeneity in the overall meta-analysis as well as in the sub-group analysis, which suggests that the underlying factors might partially contribute to the observed heterogeneity. Second, it is possible that the sample size was not large enough to generate a meaningful conclusion. Limited sample sizes are usually accompanied by selection bias. Thus, pooled results based on limited studies lack sufficient power to support or deny an association [43]. Third, in the ethnicity sub-group analyses, there was only one study that compared certain populations (African-American, Latino, Indian, and Indo-European) and only six studies that specified Caucasians. Thus, the discrepancy of association among different ethnic sub-groups should be interpreted cautiously. Finally, gene-gene interactions and epigenetic influences were not measured in our meta-analysis because of the limited availability of such information.

## Conclusions

In conclusion, our results suggest that Alu insertion into the *PGR* gene is not associated with the risk of breast cancer. More studies with larger sample sizes will be needed to validate our findings and to explore potential epigenetic mechanisms and environmental influences on the risk of breast cancer.
